# Linking Oxygen Evolution Selectivity to Water Oxidation Reaction Order on Hematite Photoanodes by Operando Photoelectrochemical Mass Spectrometry

**DOI:** 10.1021/jacs.6c11949

**Published:** 2026-09-26

**Authors:** Daniele Benetti, Soren B. Scott, Shijie Yu, James Murawski, Caiwu Liang, Anna Winiwarter, Guangmeimei Yang, Andreas Kafizas, Anna Hankin, Ifan E. L. Stephens, James R. Durrant, Flurin Eisner

**Affiliations:** † Department of Chemistry and Centre for Processable Electronics, Molecular Sciences Research Hub, Imperial College London, London W12 0BZ, U.K.; ‡ Department of Chemistry, 6396 University of Oxford, Oxford OX1 3TA, U.K.; § Department of Chemistry, University of Copenhagen, DK-2100 Copenhagen, Denmark; ∥ Department of Materials, 4615 Imperial College London, London, SW7 2AZ, U.K.; ⊥ Department of Chemical Engineering, Imperial College London, London SW7 2AZ, U.K.; # School of Engineering and Materials Science, 4617 Queen Mary University of London, London E1 4NS, U.K.

## Abstract

Water oxidation on metal-oxide (photo)­anodes is often assumed to yield molecular oxygen. Here, we combine operando photoelectrochemical mass-spectrometry oxygen detection with spectroelectrochemical rate law analyses to link oxygen selectivity to surface-hole density on hematite photoanodes. At low current and hole densities, where first-order kinetics dominate, a near-zero Faradaic efficiency for oxygen evolution is observed, revealing that isolated single-hole water oxidation is poorly coupled to molecular oxygen formation. Increasing surface-hole density sharply increases O_2_ Faradaic efficiency, showing that the kinetic crossover to cooperative third-order water oxidation is also a selectivity switch toward O_2_ evolution. Equivalent behavior under dark electrochemical operation shows that multihole chemistry is intrinsic to hematite and governs selective oxygen evolution, likely representing a general feature of semiconducting metaloxides.

## Graphical abstract

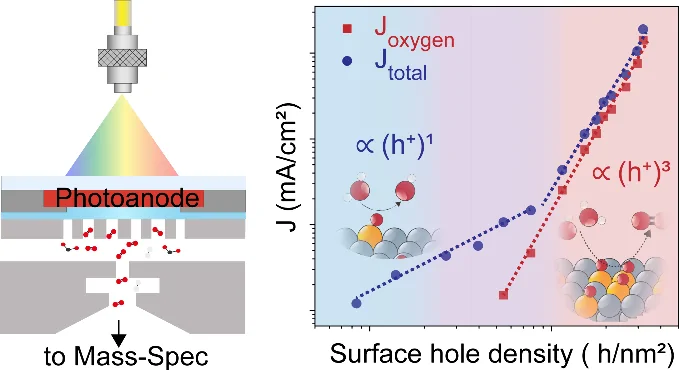

Electrochemical (EC) and photoelectrochemical (PEC) water splitting offer promising routes to green hydrogen, but are limited by the sluggish water oxidation reaction.^1,2^ Based on electrochemical measurements alone, anodic current during water oxidation is often assigned to molecular oxygen formation; indeed, the reaction is conventionally referred to as the oxygen evolution reaction (OER). However, direct *operando* quantification of O_2_ and Faradaic efficiency (FE) remains relatively limited for semiconducting metal-oxide photoanodes, especially at low reaction rates where limited detection sensitivity can obscure the true oxygen-evolution response.^3,4^ In these systems, water oxidation is governed by the accumulation of photogenerated surface holes, with the reaction rate often determined by the density of oxidizing equivalents rather than applied potential alone.^3,5–7^ As surface-hole density increases, distinct kinetic regimes have been reported, from first-order behavior at low hole density to higher-order kinetics at larger hole accumulation.^5,8,9^ However, it remains unclear whether this change in reaction order correlates with a change in oxygen evolution efficiency, with most studies to date continuing to assume that the water oxidation reaction is synonymous with oxygen evolution.

Hematite (α-Fe_2_O_3_) provides a model case for this population-driven behavior. Spectroelectrochemical studies have shown that increasing surface-hole accumulation drives a transition from first-order kinetics to a third-order regime associated with cooperative multihole catalysis.^5,6,8,10^ Under typical solar illumination, hematite operates predominantly in the third-order regime, where the rate scales with accumulated surface-hole density.^11^ However, direct molecular evidence linking this kinetic transition to the actual formation of molecular oxygen is lacking. It is not yet clear whether the low-order regime contributes significantly to O_2_ production or instead corresponds predominantly to alternative oxidation pathways such as hydroxyl-radical-like chemistry.^6,10,12,13^

Previous studies have relied on optical or transient measurements of hole populations to infer the mechanistic change, but these approaches cannot determine whether the accumulated charge effectively drives O–O bond formation and oxygen release, or drives alternative reactions. Investigating this potential change in selectivity experimentally has been challenging because photocurrent measurements alone cannot distinguish between partial and complete oxidation processes.^4,14^

Here, we address this challenge by undertaking *operando* photoelectrochemical mass-spectrometry (PEC-MS) on α-Fe_2_O_3_ photoanodes, which enables direct, real-time quantification of molecular O_2_ with pmol·s^–1^ sensitivity under operating photoelectrochemical conditions,^15^ while preserving optical access, electrochemical control, and illumination of the photoelectrode (Figure 1a). By combining this new PEC-MS capability with spectroscopic measurements of surface-hole accumulation, we correlate FE with independently determined hole-density rate laws, establishing a direct experimental link between surface charge accumulation, reaction order, and molecular oxygen formation under both photoelectrochemical and electrochemical conditions.

**Figure 1:**
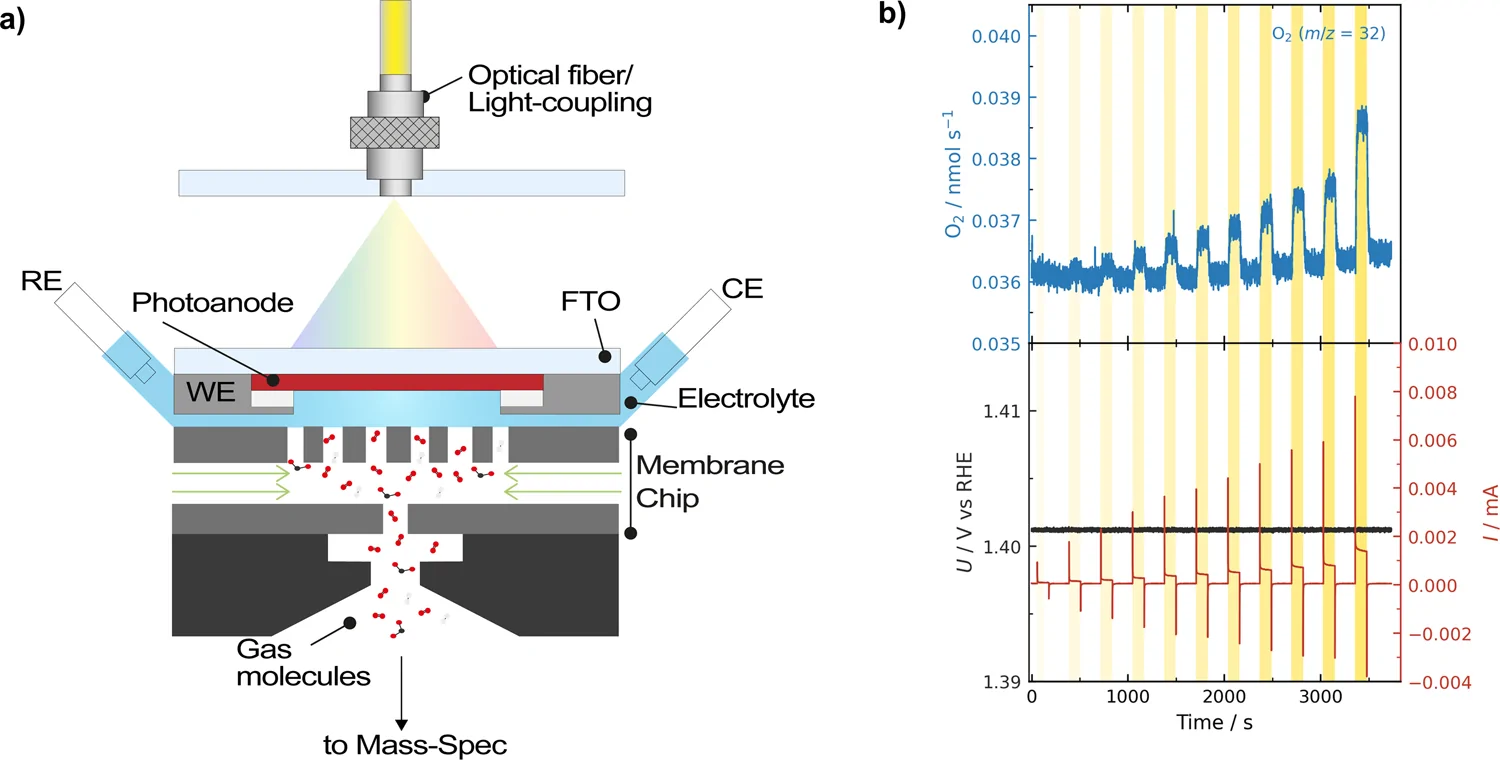
Operando PEC-MS detection of oxygen evolution from a photoanode. (a) Schematic of the specially designed PEC-MS cell, which integrates optical access, electrochemical control, and coupling to an EC-MS membrane chip for real-time detection of gas products from illuminated photoanodes (back-illumination). (b) Representative PEC-MS measurement at 1.4VRHE under modulated illumination (λ = 365 nm) (ON: 120 s, OFF: 180 s). Photocurrent and O_2_ signal are shown as a function of time. Darker shading indicates light intensity: from 0.2–1.2 mW/cm^2^ steps are 0.1 mW/cm^2^, and the last measurement is at 2.5 mW/cm^2^.

We identify a sharp threshold separating a regime with minimal oxygen formation from one in which oxygen evolution becomes the dominant anodic process. This selectivity switch coincides with the spectroscopic crossover from first- to third-order hole kinetics. The first-order regime contributes little to molecular oxygen production, indicating that it is dominated by alternative oxidation pathways, whereas efficient oxygen evolution emerges only in the cooperative multihole regime.

To establish our *operando* approach, we couple a newly designed light-compatible cell with an established EC-MS membrane-chip platform, previously limited only to electrochemical systems^15^ (Figure 1a). This configuration enables simultaneous monitoring of applied potential, photocurrent, and molecular oxygen evolution from an illuminated photoanode with subsecond (∼300 ms) response time. Figure 1b shows a representative measurement at 1.4 V_RHE_ under modulated illumination of increasing intensity. The O_2_ signal (*m*/*z* = 32) increases systematically with photon flux and follows the photocurrent response, demonstrating direct, real-time detection of molecular oxygen during photoelectrochemical operation. Potential-sweep measurements further validate the PEC-MS response (Figure S1).

To investigate how molecular oxygen evolution depends on the accumulated surface-hole population, we tuned the operating conditions (i.e., light intensity and bias) to access different kinetic regimes (Figures S2–S3). Increasing photon flux results in a proportional increase in both current and O_2_ production (Figure S3c), consistent with operation in a population-controlled (band-edge-pinned) regime.^11,16^

O_2_ FE was evaluated across a wide photocurrent range as the ratio of O_2_-associated charge to total anodic charge, integrated over the final 30s of each illumination period (see Supporting Information (SI) for details).^4^ O_2_ FE is plotted against steady-state photocurrent density, using both light-intensity-dependent measurements at fixed bias and bias-dependent measurements at fixed illumination (Figure 2). Because photocurrent in Fe_2_O_3_ scales with accumulated surface-hole density under these conditions, this representation directly relates oxygen yield to the surface-hole population and tests whether the emergence of multihole chemistry corresponds to a measurable change in oxygen selectivity.

**Figure 2:**
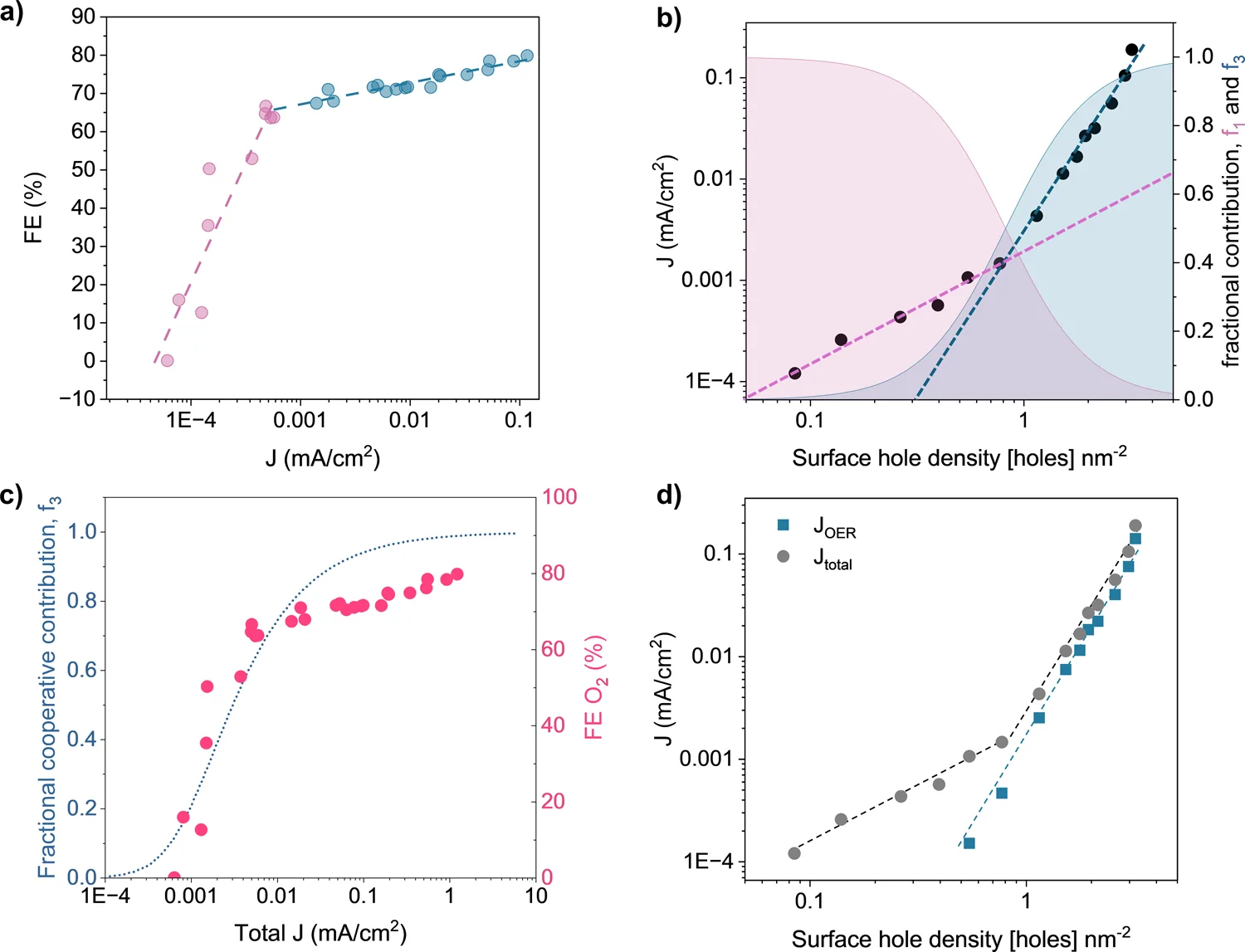
(a) FE for O_2_ evolution plotted against steady-state photocurrent density, determined from the final 30 s of each illumination period. Bias- and light-intensity-dependent measurements are combined (see SI). (b) Spectroscopically determined rate law for water oxidation on hematite at pH = 7 showing the transition from first-order (m_1_ = 1.08) to third-order kinetics (m_3_ = 3.29); shaded regions indicate fractional contributions of first-order (*f*_1_) and multihole (*f*_3_) pathways. (c) Comparison between measured FE and f_3_ as a function of current density. (d) Rate law obtained after correcting the total current by the FE to isolate J_OER_ derived from PEC-MS (see SI).

Two distinct regimes emerge in the FE-current relationship (Figure 2a). At low photocurrent densities (*J* < ∼0.6 μA·cm^–2^), FE drops sharply (reaching ∼0% at ∼0.1 μA·cm^–2^), indicating that only a fraction of extracted charge results in molecular oxygen formation. This region corresponds to the early photocurrent onset of hematite (V_ONSET_ ≈ 1.0 V_RHE,_ Figure S1), where holes accumulate predominantly in surface-localized states^16–18^ and oxidative current may be lost through recombination, as indicated by negative photocurrent transients (Figure S3),^17,19,20^ or through single-hole oxidation pathways that do not efficiently yield O_2_.^6^ Upon increasing the photocurrent density, however, the FE rises, approaching a saturation regime of ∼80%, suggesting a change in the dominant surface reaction pathway. The nonunity plateau likely reflects the specific PEC-MS measurement conditions, rather than an intrinsic limitation (see Supplementary Note 3).

A strikingly similar transition is observed in the spectroscopically determined rate law for water oxidation on hematite. Figure 2b shows the dependence of *J*_*total*_ on surface-hole density, which exhibits the characteristic crossover from first-order to approximately third-order kinetics.^8,11^

From the spectroscopic rate-law analysis, the fractional contributions of the first-order (*f*_1_) and cooperative third-order (*f*_3_) pathways can be extracted (Figure 2b). At low surface-hole densities (< 0.6 h^+^/ nm^2^), the current is dominated by the first-order pathway, whereas increasing hole accumulation leads to the emergence of the cooperative multihole regime. Strikingly, when FE is plotted together with *f*_3_ (Figure 2c), both quantities exhibit the same trend with current density: they increase sharply over the same transition range, and approach a near-saturation regime at higher reaction rates. This overlap indicates that molecular oxygen formation closely follows the emergence of the cooperative pathway described by *f*_3_, while the current associated with the *f*_1_ regime contributes predominantly to alternative oxidation processes.

This interpretation is further confirmed by analyzing the effective oxygen-evolution current, *J*_OER_. When the measured current in the spectroscopy analysis is corrected by the FE to obtain *J*_OER_, the resulting rate law follows only the third-order dependence expected for cooperative water oxidation (Figure 2d). The disappearance of the low-order component the from *J*_OER_ rate law further confirms that the first-order pathway does not significantly contribute to molecular oxygen formation.

To determine whether the relationship between multihole kinetics and O_2_ selectivity is intrinsic to hematite surface chemistry rather than specific to photoexcitation, we performed analogous measurements under dark electrochemical conditions, where surface holes are generated by anodic bias.^16,21^ The rate laws measured under illumination and in the dark overlap closely across the full surface-hole-density range (Figure 3a), indicating that the same cooperative multihole mechanism governs both photoelectrochemical and electrochemical water oxidation.

**Figure 3:**
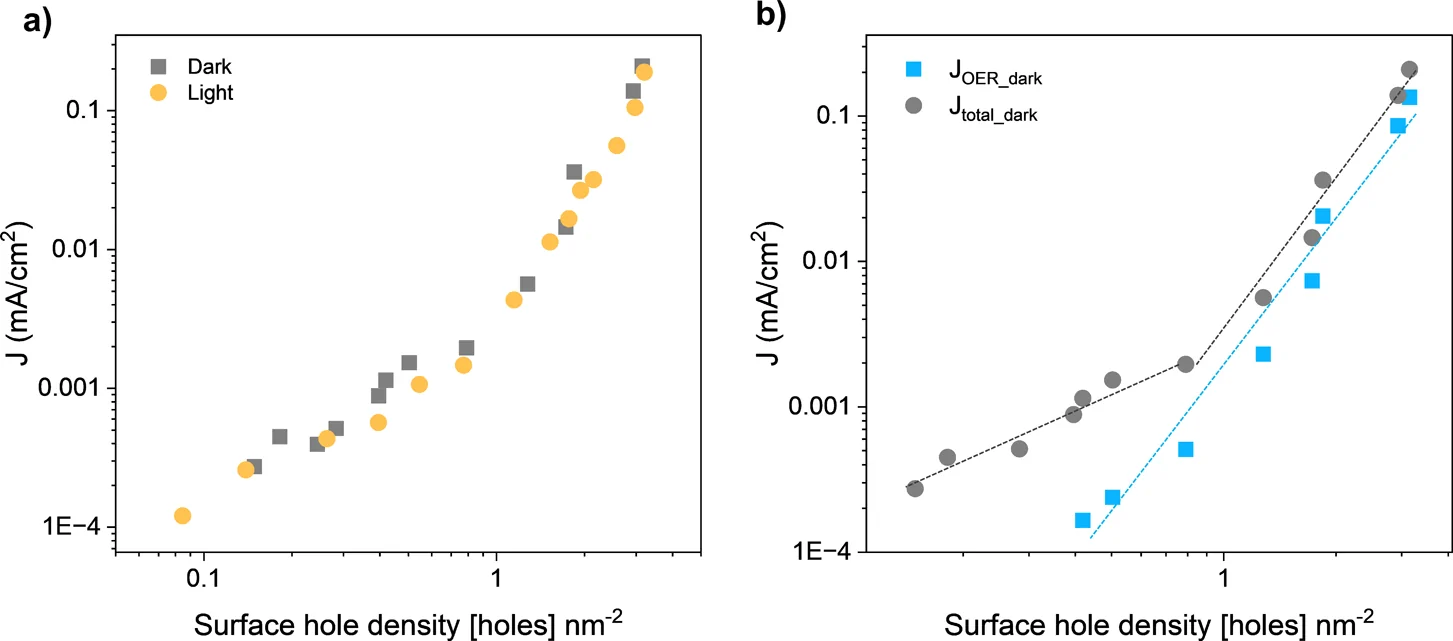
Linking oxygen selectivity to water oxidation on hematite electrodes. (a) Water-oxidation rate laws under illumination and dark electrochemical conditions, pH = 7. (b) Dark rate law after FE correction to isolate J_OER_dark_ (*J*_total_dark_: m_1_ = 1.19 and m_3_ = 3.32; J_OER_dark_: m_3_ = 3.42).

Similar kinetic behavior has previously been reported for other oxide electrodes,^21,22^ in which valenceband holes are generated in the dark via field-assisted band-to-band tunnelling (i.e., Zener mechanism) to drive water oxidation, indicating that this mechanism may be general across metal-oxide photoelectrodes.^21,23,24^

The O_2_ FE in dark is then measured as a function of current density (Figure S6a). As observed under illumination, the FE shows a transition from low to high efficiency, indicating that the fraction of charge producing molecular oxygen rises as the surface-hole population increases. Comparison with the spectroscopically derived kinetic fractions shows that this increase in O_2_ selectivity again follows the *f*_3_ pathway (Figure S6b-c), demonstrating that the same relationship between reaction order and oxygen formation is preserved under dark electrochemical conditions. We then applied the same FE correction used for the light condition to isolate the oxygen-producing current density under dark conditions, *J*_OER_dark_. The resulting rate law follows only the superlinear dependence expected for cooperative multihole oxygen evolution, with an approximately third-order dependence (Figure 3b). Thus, under dark electrochemical conditions as well, the disappearance of the low-order component in *J*_OER_dark_ shows that the first-order pathway contributes little to molecular oxygen formation.

The mechanistic picture emerging from these results is summarized schematically in Figure 4. Overall, the low FE observed in the first-order regime suggests that this pathway corresponds to oxidation processes that do not efficiently produce molecular oxygen. We have previously shown that at low surface-hole densities, holes accumulate predominantly in surface trap states,^16^ where oxidation proceeds through isolated single-hole reactions. Under these conditions, the reaction is likely limited to early oxidation steps, where oxidation of water by Fe­(IV)=O sites forms hydroxyl-radical-like intermediates that may desorb or undergo further oxidation in solution, diverting charge away from direct O_2_ formation.^6,10,12,25^ These processes are characteristic of the first-order regime and are consistent with the formation of reactive radical intermediates observed in recent measurements under similar regime.^26,27^ Because O–O bond formation does not occur directly through these isolated single-hole pathways, only a small fraction of the extracted charge results in molecular oxygen, leading to the low FE measured at low current densities.

**Figure 4:**
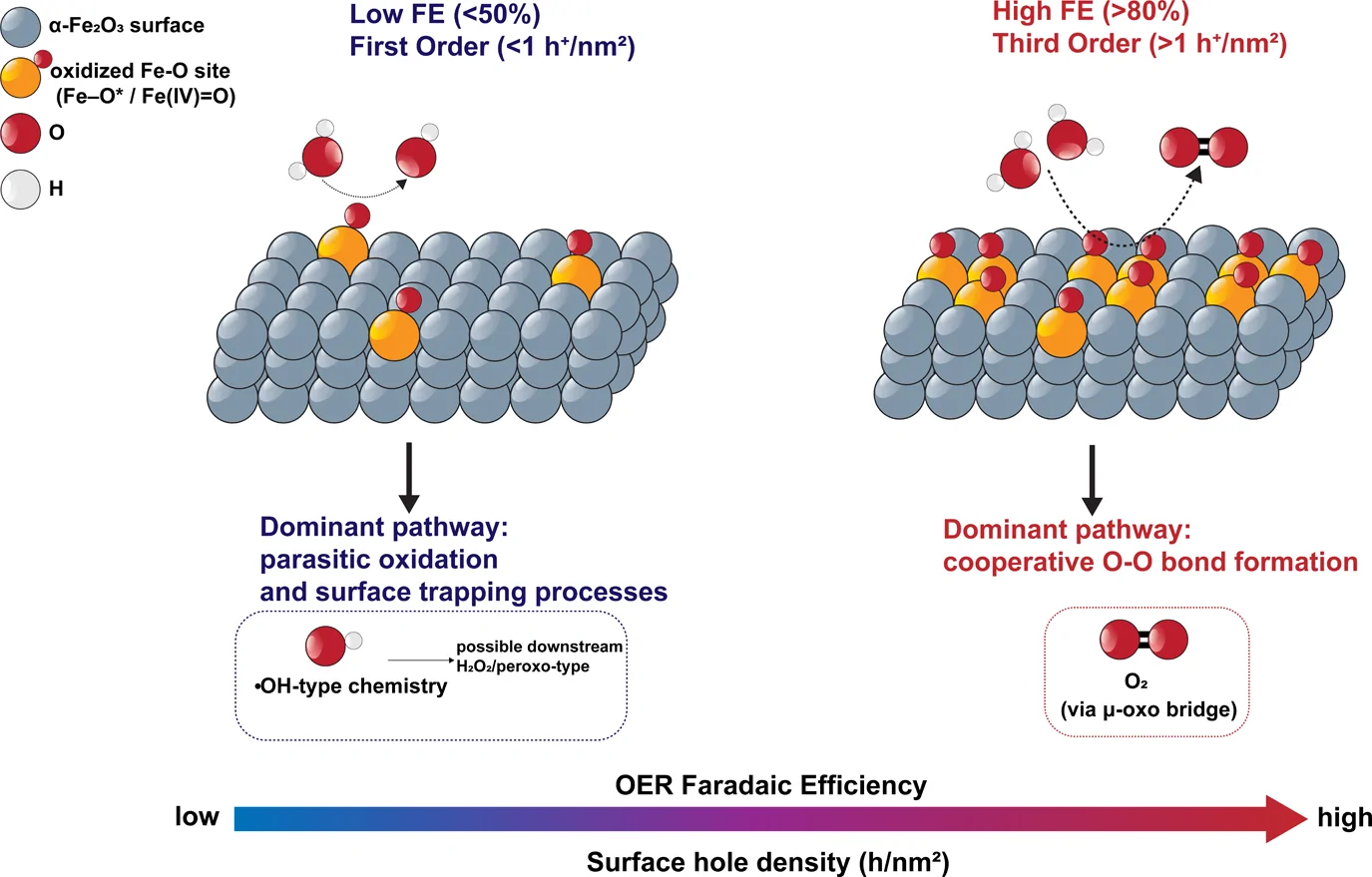
Schematic showing the correlation between FE and reaction order for water oxidation on hematite. Gray spheres represent the Fe_2_O_3_ lattice, and yellow sites denote oxidized Fe–O*/Fe­(IV)=O-like surface species, only reactive surface oxygen intermediates are shown explicitly. Low O_2_ FE coincides with first-order kinetics at low surface-hole density, consistent with limited O_2_ formation. At higher hole densities, third-order kinetics coincides with high O_2_ FE (>80%), consistent with cooperative O–O bond formation and efficient OER.

As the surface-hole population increases, these trap states become progressively filled (i.e., oxidized),^16^ allowing additional holes to accumulate in valence-band surface states capable of participating directly in the OER. In this regime, neighboring Fe–OH groups can interact cooperatively, enabling multihole chemistry characteristic of the third-order kinetic regime. Lateral coupling between adjacent surface intermediates facilitates the formation of μ-oxo-bridged species, allowing concerted O–O bond formation and efficient oxygen evolution.^10,13^ This cooperative mechanism is consistent with the spectroscopically observed transition to third-order kinetics and coincides with the region where the FE approaches its plateau value.

In summary, operando PEC-MS and the FE analysis introduced here provide the molecular counterpart to spectroscopic measurements of hole accumulation, enabling direct, real-time O_2_ quantification from a working photoanode. Combined with rate-law spectroscopy, this links charge accumulation dynamics to catalytic oxygen formation.

The close correspondence between the emergence of the cooperative multihole pathway and the increase in oxygen selectivity demonstrates that efficient O_2_ evolution on hematite arises from cooperative multihole chemistry at the oxide surface. In contrast, the first-order regime contributes substantially to the measured anodic current, especially at low light intensities/biases, but produces little molecular O_2_, consistent with competing pathways involving surface intermediates or radical species without directly forming O_2_. The FE analysis herein therefore reveals that the transition from first- to third-order water-oxidation kinetics on hematite is also a transition in oxygen-evolution selectivity. The observation that comparable kinetic behavior is preserved under photoelectrochemical and electrochemical conditions indicates that this mechanism is intrinsic to hematite surface chemistry. Similar water-oxidation rate laws reported for other metal oxides suggest that this O_2_-selectivity relationship may extend beyond hematite, although further direct measurements are required.

## Supplementary Material



## Notes

Experimental details, additional PEC-MS and spectroscopic data, calibration and control measurements, and discussion of nonunity O_2_ Faradaic efficiencies are provided in the Supporting Information. (PDF)

## References

[1] Sivula K., Van De Krol R. (2016). Semiconducting materials for photoelectrochemical energy
conversion. Nature Reviews Materials.

[2] Montoya J. H., Seitz L. C., Chakthranont P., Vojvodic A., Jaramillo T. F., Nørskov J. K. (2017). Materials for solar fuels and chemicals. Nature materials.

[3] Corby S., Rao R. R., Steier L., Durrant J. R. (2021). The kinetics of
metal oxide photoanodes from charge generation to catalysis. Nature Reviews Materials.

[4] Scott S. B., Rao R. R., Moon C., Sørensen J. E., Kibsgaard J., Shao-Horn Y., Chorkendorff I. (2022). The low overpotential
regime of acidic water oxidation part I: the importance of O 2 detection. Energy Environ. Sci..

[5] Li J., Wan W., Triana C. A., Chen H., Zhao Y., Mavrokefalos C. K., Patzke G. R. (2021). Reaction kinetics and interplay of two different surface
states on hematite photoanodes for water oxidation. Nat. Commun..

[6] Mesa C. A., Francas L., Yang K. R., Garrido-Barros P., Pastor E., Ma Y., Kafizas A., Rosser T. E., Mayer M. T., Reisner E. (2020). Multihole water oxidation catalysis
on haematite photoanodes revealed by operando spectroelectrochemistry
and DFT. Nature Chem..

[7] Zhang Y., Zhang H., Liu A., Chen C., Song W., Zhao J. (2018). Rate-Limiting O-O Bond
Formation Pathways for Water Oxidation on
Hematite Photoanode. J. Am. Chem. Soc..

[8] Le
Formal F., Pastor E., Tilley S. D., Mesa C. A., Pendlebury S. R., Grätzel M., Durrant J. R. (2015). Rate law analysis
of water oxidation on a hematite surface. J.
Am. Chem. Soc..

[9] Zhao Y., Adiyeri Saseendran D. P., Huang C., Triana C. A., Marks W. R., Chen H., Zhao H., Patzke G. R. (2023). Oxygen
evolution/reduction reaction catalysts: from in situ monitoring and
reaction mechanisms to rational design. Chem.
Rev..

[10] Righi G., Plescher J., Schmidt F.-P., Campen R. K., Fabris S., Knop-Gericke A., Schlögl R., Jones T. E., Teschner D., Piccinin S. (2022). On the origin of multihole
oxygen evolution in haematite
photoanodes. Nature Catalysis.

[11] He T., Benetti D., Tseng C., Moss B., Teschner D., Jones T. E., Kafizas A., Grätzel M., Piccinin S., Durrant J. R. (2026). Observation of Transition
from Rate
Law to Butler-Volmer Controlled Water Oxidation Kinetics on Hematite
Photoanodes. J. Am. Chem. Soc..

[12] Siahrostami S., Li G.-L., Viswanathan V., Nørskov J. K. (2017). One- or
Two-Electron Water Oxidation, Hydroxyl Radical, or H2O2 Evolution. J. Phys. Chem. Lett..

[13] Jones T. E., Teschner D., Piccinin S. (2024). Toward Realistic
Models of the Electrocatalytic
Oxygen Evolution Reaction. Chem. Rev..

[14] Kempler P. A., Nielander A. C. (2023). Reliable reporting of Faradaic efficiencies for electrocatalysis
research. Nat. Commun..

[15] Trimarco D. B., Scott S. B., Thilsted A. H., Pan J. Y., Pedersen T., Hansen O., Chorkendorff I., Vesborg P. C. (2018). Enabling real-time
detection of electrochemical desorption phenomena with sub-monolayer
sensitivity. Electrochim. Acta.

[16] Oldham, L. I. ; Benetti, D. ; Liu, T. ; Wang, D. ; Durrant, J. R. Operando spectroscopic analysis of photovoltage generation in hematite photoanodes. J. Am. Chem. Soc. 2026, 148 (24)24964 24973. 10.1021/jacs.6c04274. PMC13307358 42262481

[17] Sivula K. (2013). Metal Oxide
Photoelectrodes for Solar Fuel Production, Surface Traps, and Catalysis. J. Phys. Chem. Lett..

[18] Zhou Z., Liu J., Long R., Li L., Guo L., Prezhdo O. V. (2017). Control
of Charge Carriers Trapping and Relaxation in Hematite by Oxygen Vacancy
Charge: Ab Initio Non-adiabatic Molecular Dynamics. J. Am. Chem. Soc..

[19] Klahr B., Gimenez S., Fabregat-Santiago F., Bisquert J., Hamann T. W. (2012). Electrochemical
and photoelectrochemical investigation of water oxidation with hematite
electrodes. Energy Environ. Sci..

[20] Klahr B., Gimenez S., Fabregat-Santiago F., Hamann T., Bisquert J. (2012). Water oxidation
at hematite photoelectrodes: the role of surface states. J. Am. Chem. Soc..

[21] Li B., Oldham L. I., Tian L., Zhou G., Selim S., Steier L., Durrant J. R. (2024). Electrochemical
versus Photoelectrochemical
Water Oxidation Kinetics on Bismuth Vanadate (Photo)­anodes. J. Am. Chem. Soc..

[22] Saeed K. H., Garcia Osorio D.-A., Li C., Banerji L., Gardner A. M., Cowan A. J. (2023). Monitoring interfacial
electric fields at a hematite
electrode during water oxidation. Chemical Science.

[23] Pettinger B., Schöppel H.-R., Yokoyama T., Gerischer H. (1974). Tunnelling
Processes at Highly Doped ZnO-Electrodes in Aqueous Electrolytes Part
II: Electron Exchange with the Valence Band. Berichte der Bunsengesellschaft für physikalische Chemie.

[24] Boddy P. J. (1968). Oxygen
Evolution on Semiconducting TiO2. J. Electrochem.
Soc..

[25] Qiang Z., Chang J.-H., Huang C.-P. (2002). Electrochemical
generation of hydrogen
peroxide from dissolved oxygen in acidic solutions. Water Res..

[26] Li D., Wei R., Zhang D., Ni C., Yin H., Zhang L., Fan F., Wang X., Li C. (2025). Determining
kinetics of H2O2 evolution
from photoelectrochemical water oxidation. Nat.
Commun..

[27] Yuan X., Bender M. T., Ko M., Choi K.-S. (2025). Understanding two
voltammetric features of water reduction and water oxidation in mild
pH solutions. Nature Catalysis.

