# Supplementary material for: Linking Oxygen Evolution Selectivity to Water Oxidation Reaction Order on Hematite Photoanodes by Operando Photoelectrochemical Mass Spectrometry
Source: J Am Chem Soc. 2026 Sep 26;148(39):41739–44. doi: 10.1021/jacs.6c11949 (PMC13651520; doi:10.1021/jacs.6c11949)
Supplement: Supplementary file 1 [file ja6c11949_si_001.pdf]

Linking oxygen evolution selectivity to water oxidation reaction order on hematite photoanodes by operando photoelectrochemical mass spectrometry

Daniele Benetti[1,2,*], Soren B. Scott[3], Shijie Yu[2], James Murawski[4], Caiwu Liang[1,4], Anna Winiwarter[4], Guangmeimei Yang[1], Andreas Kafizas[1], Anna Hankin[5], Ifan E. L. Stephens[4], James R. Durrant[2], Flurin Eisner[4,6*]

[1] Department of Chemistry and Centre for Processable Electronics, Molecular Sciences Research Hub, Imperial College London, London W12 0BZ, UK.

[2] Department of Chemistry, University of Oxford, Oxford OX1 3TA, UK.

[3] Department of Chemistry, University of Copenhagen, Copenhagen, Denmark

[4] Department of Materials, Imperial College London, London, SW7 2AZ, UK.

[5] Department of Chemical Engineering, Imperial College London, London SW7 2AZ, UK

[6] School of Engineering and Materials Science, Queen Mary University of London, London E1 4NS, UK.

* Corresponding authors
Daniele Benetti, d.benetti@imperial.ac.uk; daniele.benetti@chem.ox.ac.uk
Flurin Eisner, f.eisner@qmul.ac.uk

SUPPLEMENTARY INFORMATION

1 Experimental Section 2
1.1 Hematite Film Preparation 2
1.2 Structural and Morphological Characterization 2
1.3 Electrochemical and Photoelectrochemical Measurements 2
1.4 Photoelectrochemistry–Mass Spectrometry (PEC-MS) 3
1.5 PEC-MS Measurement Procedure for Oxygen Detection 3
1.6 Photoinduced Absorption Spectroscopy (PIA) 7
1.7 Step Potential Spectroelectrochemistry (SP-SEC) 7
2 Deconvolution of first- and third-order kinetic contributions and determination of oxygen-evolution current 8
3 Discussion of Faradaic Efficiency Values for Hematite Photoanodes 9
4 Comparison Between Cappuccino Cell and PEC-MS Cell 10

1 Experimental Section

1.1 Hematite Film Preparation

Hematite (α-Fe_2O_3) photoanodes were prepared by a solvothermal synthesis followed by thermal conversion of FeOOH.[1] Fluorine-doped tin oxide (FTO) glass substrates were first cleaned by sequential sonication in acetone, isopropanol, and deionized (DI) water, followed by drying under a nitrogen stream. FeOOH precursor films were grown on the FTO substrates from an aqueous solution containing 0.15 M iron(III) chloride hexahydrate ($FeCl_3$ $6H_2O$, 97%, Alfa Aesar) and 1 M sodium nitrate ($NaNO_3$, 99%, Alfa Aesar) prepared in 100 mL of DI water. The substrates were placed in a sealed solvothermal reactor and the reaction was carried out at 100 °C for 1 h. To confine the growth of the film to the front side of the FTO substrate, Kapton tape must be applied to cover the backside of the substrate during the solvothermal growth step. After growth, the films were rinsed thoroughly with DI water and dried under a nitrogen stream.

The FeOOH precursor films were subsequently converted to hematite by calcination at 800 °C for 5 min in air. The Kapton tape was removed prior to the calcination step. The resulting hematite films had a nominal thickness of approximately 30–50 nm and a surface roughness factor of ~4, consistent with previous reports for solvothermally prepared hematite photoanodes.[1,2]

1.2 Structural and Morphological Characterization

X-ray diffraction (XRD) patterns were recorded using a PANalytical X'Pert PRO diffractometer equipped with a Cu Kα radiation source (λ = 1.54 Å). (Figure S5)

Scanning electron microscopy (SEM) images were collected using a field-emission scanning electron microscope (Zeiss Auriga, accelerating voltage of 5 kV) to evaluate the morphology and surface structure of the hematite films. (Figure S5)

1.3 Electrochemical and Photoelectrochemical Measurements

Electrochemical (EC) and photoelectrochemical (PEC) measurements were carried out in a three-electrode configuration using the hematite-coated FTO substrate as the working electrode, a Pt wire as the counter electrode, and an Ag/AgCl (saturated KCl) electrode as the reference. All potentials were converted from $V_{Ag/AgCl}$ to the reversible hydrogen electrode (VRHE) scale using the Nernst equation. The electrolyte consisted of 1 M KPi buffer at pH 7.2, prepared using ultrapure water and contained no intentionally added chloride. (Millipore, >18.2 MΩ·cm). The Ag/AgCl reference electrode was housed in a separate double-junction glass chamber and was not in direct contact with the working-electrode compartment. The internal reference-electrode filling solution was separated from the working electrolyte by the junction of the reference electrode and by a second porous frit at the end of the external glass chamber, thereby strongly limiting chloride transport into the PEC-MS working compartment.

Current–voltage (J–V) curves were recorded at a scan rate of 0.001 V s^{-1} and 0.02 V s^{-1}. For PEC measurements performed in the spectroscopy setups, illumination was provided by a 365 nm LED light source, while the potential was controlled using an Autolab PGSTAT101 potentiostat. A 365 nm LED with a light intensity of approximately 12 mW cm^{-2} produced a photocurrent response comparable to that obtained under 1 sun illumination for the hematite photoanodes used in this study. All potentiostats were operated using NOVA software.

Broadband-light test. Additional PEC-MS measurements were performed under broadband white-light illumination (420–900 nm, 90 mW cm^{-2}) while varying the applied potential. The O_2 Faradaic efficiency showed the same qualitative evolution observed under 365 nm excitation, increasing from negligible values at low bias/current density to approximately 80% at higher current density. This indicates that the transition in O_2 selectivity is not specific to monochromatic 365 nm excitation and is not an artefact of highly localised, surface-confined excitation.

1.4 Photoelectrochemistry–Mass Spectrometry (PEC-MS)

Photoelectrochemistry–mass spectrometry (PEC-MS) measurements were performed using an interface system purchased from Spectro Inlets A/S. The experimental configuration is based on previously reported designs.[3,4]

A custom 3D-printed photoelectrochemical cell (from redox.me) was used, allowing illumination of the backside of the hematite electrode using LED light sources. The hematite samples were prepared with dimensions of 7.5 mm × 7.5 mm. Due to the geometry of the working electrode interface, the effective electrochemically active area was approximately 0.3 cm^2.

The EC-MS interface consists of a silicon-on-insulator microchip containing an internal cavity that acts as a microscopic headspace. This cavity equilibrates with the electrolyte working volume and allows volatile electrochemical products to be transferred directly to the vacuum chamber of the mass spectrometer without the need for differential pumping. The mass spectrometer consisted of a QMG electronics system coupled with a QMA 125 mass analyzer (Pfeiffer Vacuum). Electrochemical control during EC-MS measurements was provided by a Biologic SP-150 potentiostat. To stabilize the electrode potential during EC-MS measurements, the working electrode was connected through a 100 Ω series resistor. At the current levels relevant to this study (typically on the order of microamperes), the resulting potential drop across the resistor was negligible and did not significantly affect the applied potential. The electrolyte used during PEC-MS measurements was 1 M KPi buffer, pH 7.2, prepared using high-purity deionized water (Millipore, >18.2 MΩ·cm). The scan rate for JV curves was 1mV/s.

The PEC-MS measurements were performed using a static electrolyte volume; the electrolyte was not circulated through the cell. The electrolyte was initially air-saturated. After filling the cell, the system was allowed to equilibrate until a stable m/z = 32 background was obtained. The local background immediately preceding each measurement was used as the baseline, and photoelectrochemically generated O_2 was quantified from the increase in the calibrated m/z = 32 signal above this background. Representative raw traces are shown in Figure S2.

O_2 generated at the hematite surface enters the dissolved phase and is continuously sampled by the membrane-based PEC-MS interface. Dissolved O_2 is transported through the electrolyte to the membrane, enters the internal microchip cavity acting as a microscopic headspace, and is subsequently transferred to the mass spectrometer.[3,4]

1.5 PEC-MS Measurement Procedure for Oxygen Detection

Oxygen evolution during photoelectrochemical water oxidation was monitored using electrochemistry–mass spectrometry (EC-MS) by recording the signal at m/z = 32

corresponding to molecular oxygen. The background-corrected m/z = 32 signal was converted into a molar O_2 flux using calibration with a Pt electrode in the same PEC-MS configuration. The O_2 Faradaic efficiency was calculated from the steady-state O_2 flux and current averaged over the final 30 s of each measurement period, after the transport transient had stabilised, following previously reported methodology.[3,4]

The measurements were performed using potential or illumination step protocols in order to clearly resolve the oxygen evolution signal and allow the system to return to baseline between measurement periods (Figure 1, Figure S2, Figure S5). The Faradaic-efficiency dataset was compiled from multiple independent measurement sequences using different acquisition protocols and was therefore not obtained only from a single continuously increasing potential or illumination-intensity series. In some measurements, the electrode was returned to a common lower potential between successive anodic steps, while in others the current density was varied through discrete illumination steps at fixed bias or through discrete potential steps at fixed illumination. Consequently, individual current-density regimes were accessed with different preceding potential and illumination histories. No systematic dependence on the acquisition sequence was observed. Importantly, data obtained using these different protocols converge onto the same relationship between O_2 Faradaic efficiency and steady-state current density across the investigated range. No systematic dependence on the acquisition sequence was observed. This reproducibility indicates that the observed Faradaic-efficiency trend is primarily associated with the steady-state current density and accumulated surface-hole population, rather than with the particular experimental parameter or measurement history used to reach that condition.

O2 Calibration: The O_2 sensitivity factor of the PEC-MS was calibrated using oxygen generated via the electrochemical oxygen evolution reaction (OER) on a polycrystalline Pt electrode (5 mm diameter, 99.99%, Pine Research Instrumentation, Inc.) in 0.1 M $HClO_4$. The Pt electrode was pretreated by holding the potential at 1.6 VRHE for 30 min to form a surface oxide layer, ensuring 100% Faradaic efficiency for oxygen evolution. Chronopotentiometry was then performed at current densities ranging from 50 to 500 µA cm^{-2}, with each current applied for 10 min. A linear relationship between the MS signal and the calculated O_2 generation rate was obtained, and the slope of this correlation was used to determine the sensitivity factor of the PEC-MS.

Two main experimental approaches were used:

i) *Fixed illumination with stepped applied potential*

In these experiments, the light intensity was kept constant while the applied potential was varied in discrete steps. The potential was typically stepped in increments of 25-50 mV (for example from 1.00 V_{RHE} to 1.05V_{RHE} and then returned to the initial potential).

Each potential step was maintained for sufficient time to obtain a stable current and O_2 signal, followed by a recovery period before the subsequent measurement. For standard measurements, the potential was held at the target value for 120 s under illumination, followed by 180 s resting period. The same step protocol was repeated sequentially for each applied potential.

Dark electrochemical measurements were performed using the same potential stepping protocol, in the absence of illumination.

ii)   Fixed applied potential with stepped illumination intensity

In a second measurement mode, the applied potential was kept constant while the illumination was modulated using the LED light source. In this case, the light intensity was varied between measurement cycles while maintaining the same timing protocol. Different illumination intensities were investigated over multiple sequences rather than exclusively as a single monotonic intensity progression, with dark recovery periods between illumination cycles.

For these measurements, the illumination was applied for 120 s followed by 180 s in the dark to allow stabilization of both the photocurrent and the mass spectrometry signal.

At higher illumination intensities, longer pulse durations were used to improve signal stability. In these cases, the illumination period was increased to 180 s, followed by 270 s in the dark.

This longer cycle was implemented because higher photocurrent densities can lead to oxygen bubble formation at the electrode surface, which can temporarily disturb the mass spectrometry signal and lead to fluctuations in the measured oxygen flux. The extended dark period allows the system to re-equilibrate before the next measurement cycle.

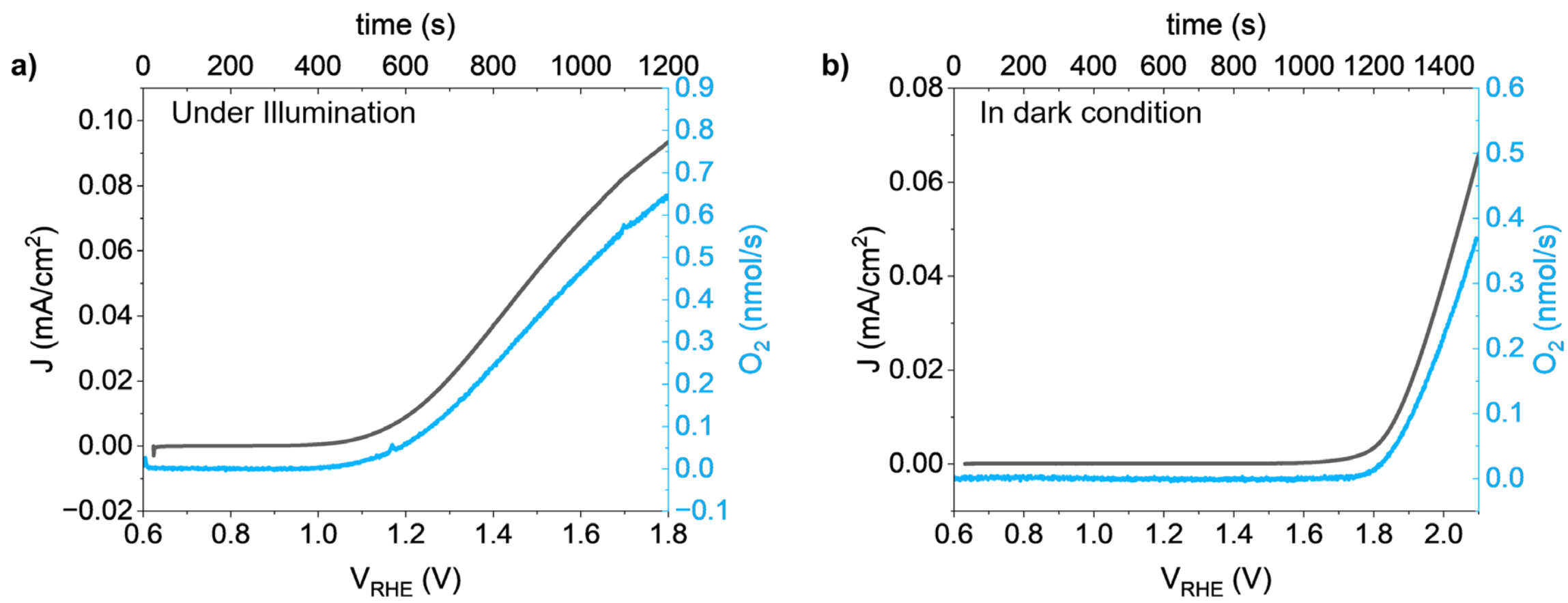

Figure S1: (a) Linear sweep measurement showing photocurrent density and simultaneously detected O_2 evolution recorded under 10.8 mW/cm^2, 365 nm radiation. (b) Dark electrochemical measurement showing current density and O_2 evolution as a function of applied potential.

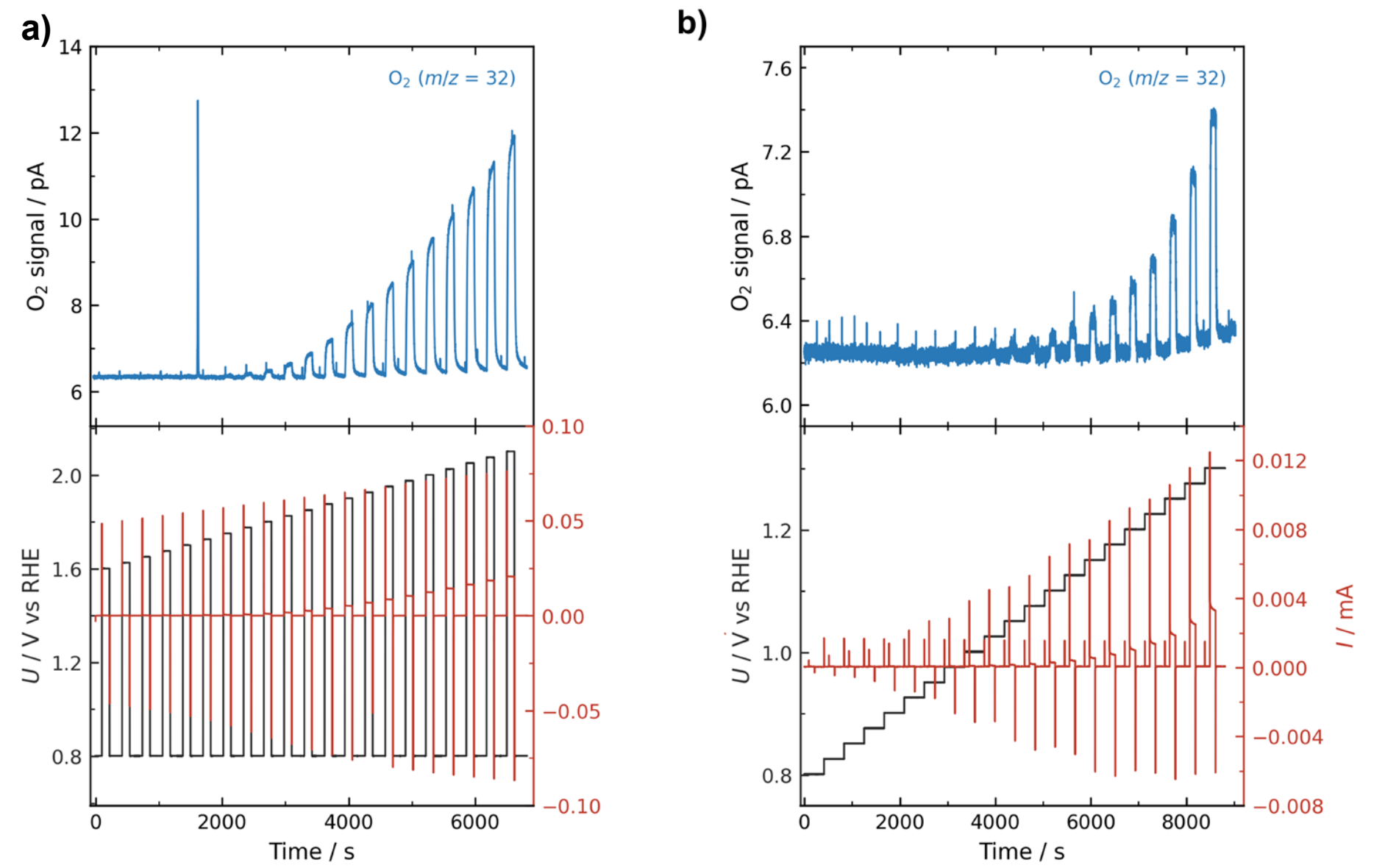

Figure S2: Representative operando PEC-MS raw measurements on hematite photoanodes in 1 M KP buffer (pH 7.2). All panels show the simultaneously detected O_2 signal (m/z = 32, top) and the applied potential (black, left axis) with the measured current (red, right axis) as a function of time (bottom). (a) Dark electrochemical measurements. The potential was stepped from 1.6 to 2.1 V vs RHE in increments of 25 mV, with each step held for 120 s followed by a 180 s recovery at 0.8 V vs RHE. (b) PEC-MS measurements under constant illumination (10.8 mW/cm^2). The potential was stepped from 0.8 to 1.3 V vs RHE in 25 mV increments, with each step held for 300 s.

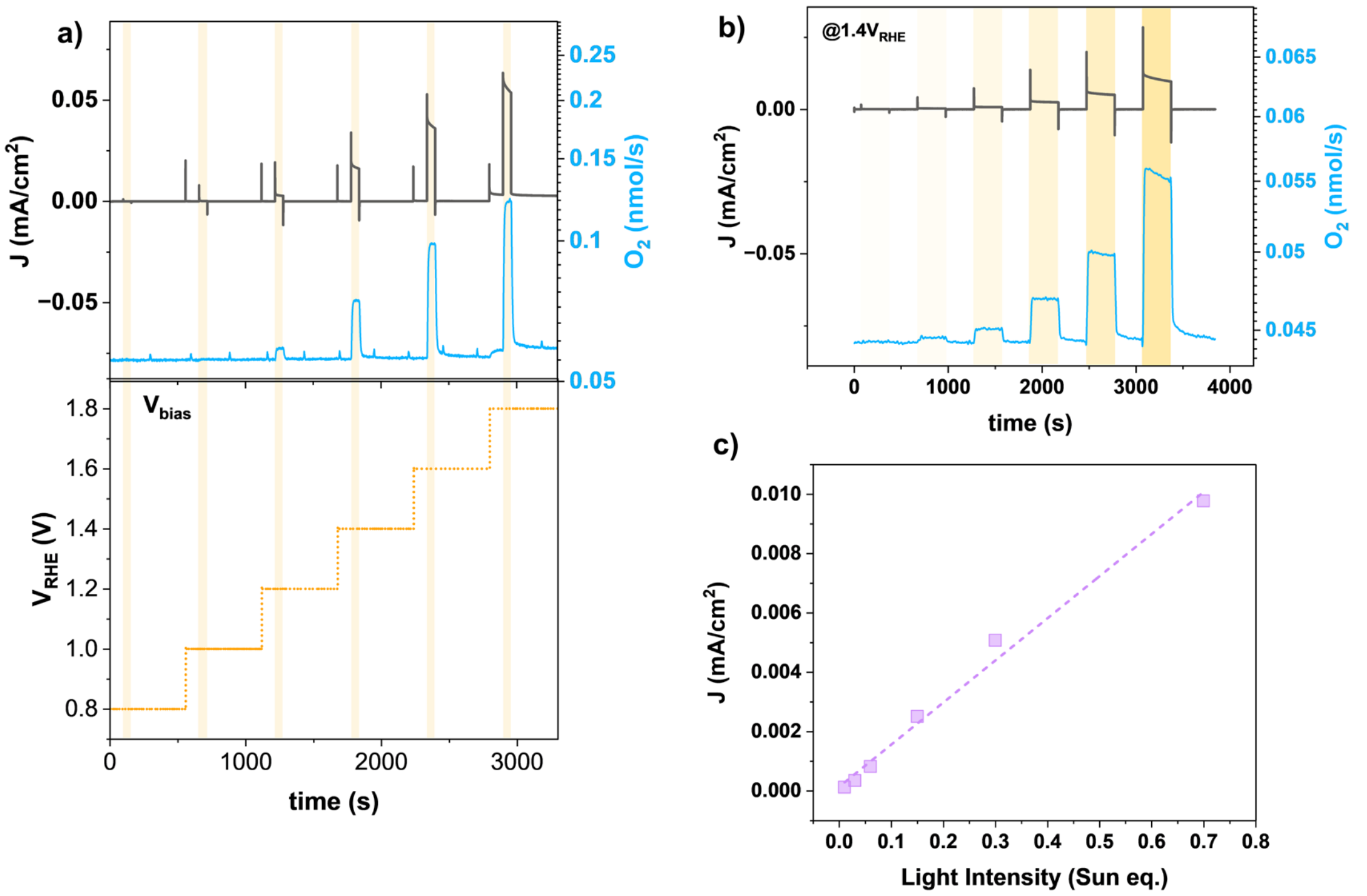

Figure S3. (a) Simultaneous measurement of photocurrent density and oxygen evolution during stepwise changes in applied bias under constant illumination (10.8 mW/cm^2). The lower panel shows the corresponding applied potential profile. (b) Calibrated oxygen evolution measured at a fixed bias (1.4 VRHE) while varying the illumination intensity from 0.18 to 10.2 mW/cm^2. (c) Steady-state photocurrent density (@1.4 VRHE) as a function of light intensity, showing the increase in reaction rate with increasing photogenerated hole flux.

1.6 Photoinduced Absorption Spectroscopy (PIA)

Photoinduced absorption (PIA) spectroscopy measurements were performed using a home-built transient absorption system. The probe light source was a tungsten–halogen lamp (Osram 6460 HLX bulb) housed in a Bentham IL1 lamp housing and powered by a Bentham 610 power supply. A series of focusing and collimating lenses were used to direct the probe light onto the sample and subsequently into the detector. After passing through the sample, the probe light was dispersed using a monochromator (OBB) in order to select the desired detection wavelength. The transmitted probe light was detected using a silicon photodiode detector (Hamamatsu) coupled to an amplifier module (Costronics). The resulting change in optical density (ΔO.D.) was recorded using a data acquisition card (NI USB-6361).

The electrochemical bias applied to the sample was supplied by a potentiostat (Autolab PGSTAT101), which simultaneously recorded the photocurrent generated in response to the pump illumination. The current signal was recorded using a digital oscilloscope (Tektronix DPO 3012) and subsequently converted into current density values. The potentiostat was controlled using NOVA software.

Excitation of the sample was achieved using a 365 nm LED, powered by a power supply. The LED was triggered electronically using a MOSFET (ZVN4306A). During the measurements, the LED was operated with a 10 s illumination period followed by a 20 s dark period. Typically 10–20 signal averages were collected for each measurement. For measurements performed at low light intensities, up to 75 averages were used to improve the signal-to-noise ratio. For measurements where background correction is required, a B-test can be performed by inserting a shutter to block the probe light from reaching both the sample and the detector. The resulting background signal is then subtracted from the corresponding A-test measurement to obtain the final optical signal. Initial control experiments confirmed that no detectable photoluminescence from the sample contributed to the signal, and therefore all subsequent measurements were performed without applying the B-test correction. The entire spectroscopy system was controlled using custom LabVIEW software, which synchronized the measurement sequence by triggering the DAQ card, oscilloscope, and LED driver at the start of each measurement.

1.7 Step Potential Spectroelectrochemistry (SP-SEC)

Step potential spectroelectrochemistry (SP-SEC) measurements were performed using the same optical spectroscopy setup described for the PIA experiments, with the electrochemical potential step used as the excitation stimulus instead of optical illumination. The applied potential was controlled using the Autolab PGSTAT101 potentiostat, operated through NOVA software. A square-wave potential program was applied to the electrode, consisting of three sequential steps: the system was held at an initial potential V_0 for 5 s, stepped to V_1 for 15 s, and then returned to V_0 for 15 s. A trigger output from the potentiostat was used to synchronize the optical acquisition by directly triggering the DAQ card and oscilloscope. The resulting optical response and current transient were recorded using the same detection configuration described for the PIA measurements.

2 Deconvolution of first- and third-order kinetic contributions and determination of oxygen-evolution current

To quantify the relative contributions of single-hole and cooperative multihole oxidation pathways, the total steady-state current density (J_{tot}) can be expressed as the sum of two contributions:

$$J(A) = J_1(h) + J_3(h)$$

where *A* is the accumulated surface hole population obtained from converting photoinduced absorption (PIA) signal. The terms J_1 and J_3 represent the current contributions associated with first-order and third-order kinetics, respectively. Each contribution can be described using a power-law dependence:

$$J_1(A) = 10^{b_1} A^{m_1}$$
$$J_3(A) = 10^{b_3} A^{m_3}$$

Where b_1 and b_3 are the intercepts and m_1 and m_3 are the slopes obtained from linear fits to the low- and high-hole-density regions of the $\log J$ versus $\log A$ plots. The extracted slopes were found to be $m_1 \approx 1.08$ and $m_3 \approx 3.29$, consistent with first-order and cooperative third-order kinetics, respectively.
Using these fitted expressions, the individual current contributions can be reconstructed over the full range of *A*, allowing the fractional contribution of each pathway to be determined as :

$$f_1(A) = \frac{J_1}{J_{\mathrm{tot}}}, f_3(A) = \frac{J_3}{J_{\mathrm{tot}}}$$

with $J_{\mathrm{tot}} = J_1 + J_3$, such that $f_1 + f_3 = 1$ at each value of A.

This decomposition enables direct visualization of the transition from single-hole oxidation processes dominating at low surface hole densities to cooperative multihole pathways at higher hole populations.

The oxygen-evolving current density J_{OER} can be obtained by combining the total current density J_{tot} from the rate-law analysis with the FE measured by operando PEC–MS.

Because FE is determined at discrete current values, the FE–current relationship was interpolated to estimate FE(J_{tot}). The interpolation was performed only within the range of experimentally measured FE values to avoid extrapolation artifacts.

The interpolated FE values can be then used to calculated J_{OER} as:

$$J_{OER} = \frac{FE(J_{tot})}{100} \times J_{tot}$$

3 Discussion of Faradaic Efficiency Values for Hematite Photoanodes

The measured Faradaic efficiency for oxygen evolution increases with increasing photocurrent density but remains below 100% under the experimental conditions investigated in this study, even at higher photocurrent densities where cooperative multihole mechanisms dominate. Several factors may contribute to this deviation from unity Faradaic efficiency.

The present PEC-MS configuration selectively detects volatile products and therefore does not directly identify non-volatile soluble products or short-lived surface intermediates that may contribute to the non-O_2 current. In particular, H_2O_2 cannot be quantified directly using the present membrane-based MS configuration. Previous studies have proposed peroxide-type pathways in the low-current, first-order kinetic regime of hematite.[5] More recent work has directly demonstrated first-order H_2O_2 evolution with respect to surface-hole density on $BiVO_4$.[6] These observations support peroxide-type pathways as a plausible contribution to the non-O_2 current; however, the identity of the competing products is not established in the present study.

The analysis of the PEC-MS photocurrent transients (Figure 1b in the main text) reveals negative current spikes upon illumination chopping, which are indicative of significant charge recombination processes at the electrode surface.[7–9] The presence of these recombination transients even at relatively high applied bias suggests that under the PEC-MS measurement conditions, a fraction of the photogenerated holes recombine before contributing to oxygen evolution. Such recombination can influence the population of holes available for interfacial oxidation and may affect competition between the different surface pathways.[10,11]

The recombination observed in the PEC-MS measurements likely arises from the combined effects of back illumination and the electrolyte conditions used in the EC-MS setup. The PEC-MS experiments were performed using back illumination of the hematite electrode. In this configuration, photogenerated holes are generated closer to the FTO substrate and must diffuse through the hematite film to reach the semiconductor–electrolyte interface. This transport pathway can increase the probability of electron–hole recombination relative to front illumination configurations.[12–14]

Additionally, the PEC-MS measurements were performed in a near-neutral electrolyte (pH 7.2) due to compatibility requirements of the EC-MS microchip interface. Hematite photoanodes typically exhibit improved water oxidation kinetics and reduced recombination under strongly alkaline conditions.[15] At neutral pH, the slower water oxidation kinetics can increase the residence time of surface holes and intermediates, thereby increasing the likelihood of recombination and of competing pathways.[15,16]

Finally, small systematic uncertainties in PEC-MS calibration, gas collection, background subtraction, and current integration may also contribute to the absolute Faradaic-efficiency value. These uncertainties are unlikely to affect the central trend, namely the sharp increase in O_2 selectivity with increasing photocurrent and surface-hole density.

Taken together, these observations indicate that several processes may contribute to the residual non-O2 current, including possible competing oxidation pathways and some systematic uncertainties in the PEC-MS measurement. Their individual contributions cannot be resolved from the present experiments. These observations are nevertheless consistent with the mechanistic interpretation presented in this work, where increasing surface hole populations promote the transition from single-hole oxidation pathways toward cooperative

multi-hole O–O bond formation, leading to increased oxygen selectivity at higher photocurrent densities.

4 Comparison Between Cappuccino Cell and PEC-MS Cell

For direct comparison between the PEC-MS measurements and the spectroscopically derived rate-law analysis, the current density axis obtained from the PEC-MS experiments was rescaled by a constant multiplicative factor. This normalization accounts for systematic differences in the measured current density arising from variations in electrode geometry, illumination configuration, and electrochemical cell design between the PEC-MS setup and the conventional photoelectrochemical ("cappuccino") cell used for the spectroscopic measurements.

In particular, the PEC-MS measurements are performed using a confined microcell geometry with a restricted electrochemically active area (~0.3 cm^2) and back illumination, whereas the spectroscopic rate-law analysis is based on measurements performed in a standard PEC configuration with a larger illuminated area (~1.9 cm^2) and different light absorption profile. These differences lead to a systematic reduction in the measured current density in the PEC-MS setup compared to the conventional PEC cell (Figure S4).

To account for these effects, a constant scaling factor (x8.2) was applied to the PEC-MS current density. This factor was determined by comparing the steady-state current densities measured under similar nominal operating conditions (applied potential and illumination intensity) in the two setups. Specifically, the current density ratio was calculated at four representative bias points, and the final scaling factor was taken as the average of these values. This approach provides a robust estimate of the systematic difference in current magnitude between the two setups while preserving the underlying dependence of the current on surface hole density. Importantly, this normalization affects only the absolute magnitude of the current density and does not alter the shape of the current dependence, the position of the kinetic transition, or the underlying mechanistic interpretation. The same scaling factor was applied consistently across all PEC-MS datasets used in the comparison. The excellent agreement observed after normalization between the Faradaic efficiency evolution and the spectroscopically derived fractional contribution of the cooperative pathway (f_3) (Figure 2c) supports the validity of this approach and indicates that the scaling reflects experimental differences between setups rather than intrinsic changes in reaction mechanism.

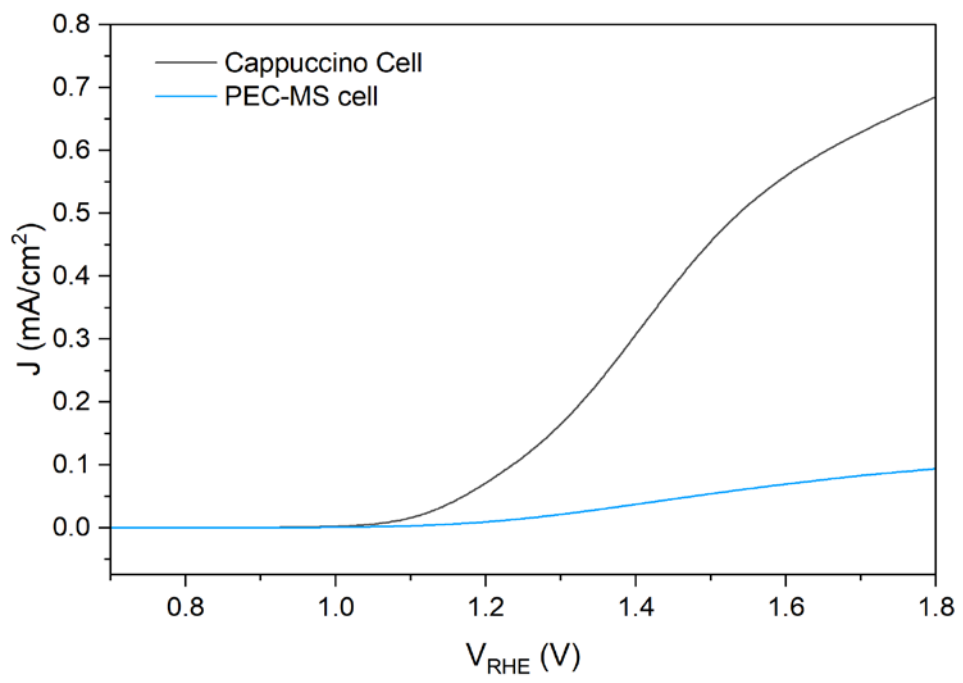

Figure S4. Comparison of photocurrent density–potential (J–V) curves for hematite photoanodes measured in a conventional "cappuccino" cell (under 100 mW/cm^2, AM1.5G) and a PEC-MS cell under back illumination (10mW/cm^2, 365 nm).

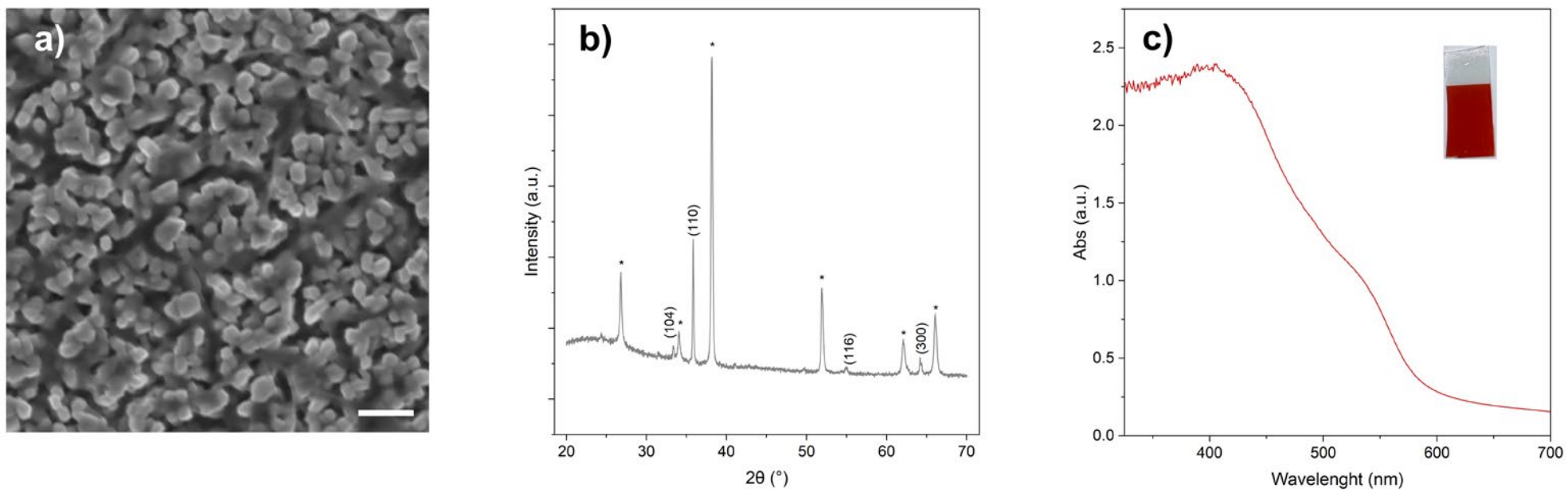

Figure S5. (a) Scanning electron microscopy (SEM) image of representative Fe_2O_3 used in this work. Scale bar, 500 nm. (b) X-ray diffraction (XRD) pattern of the hematite (α-Fe_2O_3) thin film deposited on an FTO substrate. Peaks marked with an asterisk (*) are assigned to the underlying FTO (fluorine-doped tin oxide) substrate. (c) UV–vis absorption spectrum of a representative photoanode deposited on FTO. The inset shows a photograph of the corresponding sample. The observed morphology and crystal structure are consistent with previous reports.[1,2]

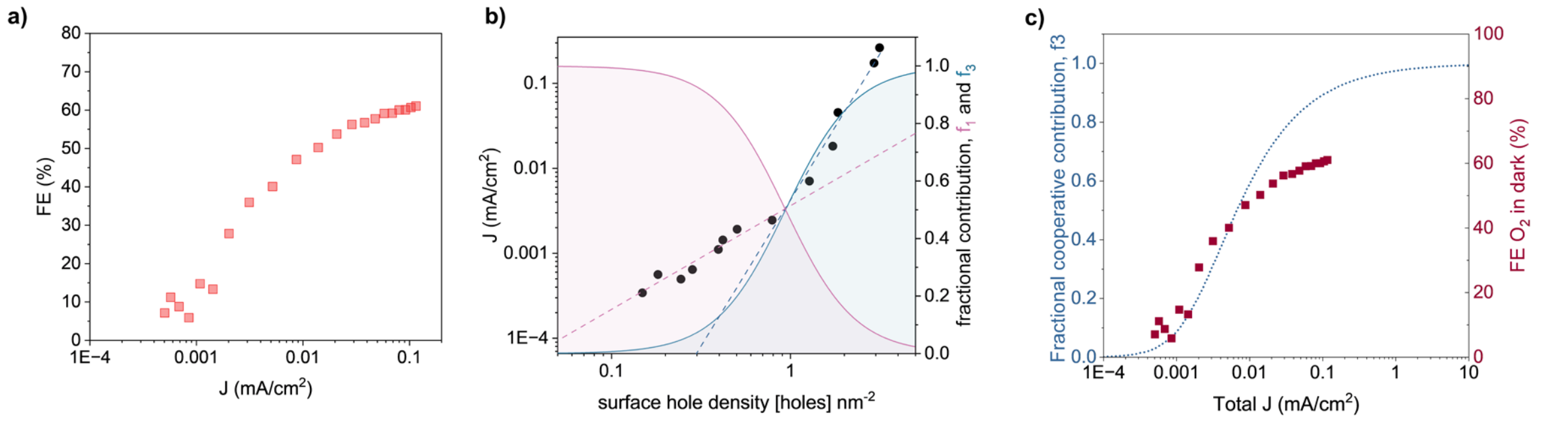

Figure S6. Dark operando analysis of oxygen evolution on hematite. (a) FE for oxygen evolution as a function of steady-state current density measured under dark conditions. (b) Spectroscopically determined rate law for water oxidation on hematite at pH 7 in the dark, showing the transition from first-order to third-order kinetics; shaded regions indicate the fractional contributions of the first-order (f_1) and multihole (f_3) pathways. (c) Comparison between the measured FE in the dark and the fractional contribution of the cooperative pathway (f_3) as a function of current density.

REFERENCES

(1) Jang, J.-W.; Du, C.; Ye, Y.; Lin, Y.; Yao, X.; Thorne, J.; Liu, E.; McMahon, G.; Zhu, J.; Javey, A.; Guo, J.; Wang, D. Enabling Unassisted Solar Water Splitting by Iron Oxide and Silicon. *Nat. Commun.* **2015**, *6* (1), 7447. https://doi.org/10.1038/ncomms8447.

(2) Oldham, L. I.; Benetti, D.; Liu, T.; Wang, D.; Durrant, J. R. Operando Spectroscopic Analysis of Photovoltage Generation in Hematite Photoanodes. **2026**. https://doi.org/10.26434/chemrxiv.15000384/v1.

(3) Trimarco, D. B.; Scott, S. B.; Thilsted, A. H.; Pan, J. Y.; Pedersen, T.; Hansen, O.; Chorkendorff, I.; Vesborg, P. C. K. Enabling Real-Time Detection of Electrochemical Desorption Phenomena with Sub-Monolayer Sensitivity. *Electrochimica Acta* **2018**, *268*, 520–530. https://doi.org/10.1016/j.electacta.2018.02.060.

(4) Scott, S. B.; Rao, R. R.; Moon, C.; Sørensen, J. E.; Kibsgaard, J.; Shao-Horn, Y.; Chorkendorff, I. The Low Overpotential Regime of Acidic Water Oxidation Part I: The Importance of O 2 Detection. *Energy Environ. Sci.* **2022**, *15* (5), 1977–1987. https://doi.org/10.1039/d1ee03914h.

(5) Mesa, C. A.; Francàs, L.; Yang, K. R.; Garrido-Barros, P.; Pastor, E.; Ma, Y.; Kafizas, A.; Rosser, T. E.; Mayer, M. T.; Reisner, E.; Grätzel, M.; Batista, V. S.; Durrant, J. R. Multihole Water Oxidation Catalysis on Haematite Photoanodes Revealed by Operando Spectroelectrochemistry and DFT. *Nat Chem* **2020**, *12* (1), 82–89. https://doi.org/10.1038/s41557-019-0347-1.

(6) Li, D.; Wei, R.; Zhang, D.; Ni, C.; Yin, H.; Zhang, L.; Fan, F.; Wang, X.; Li, C. Determining Kinetics of H2O2 Evolution from Photoelectrochemical Water Oxidation. *Nat. Commun.* **2025**, *16* (1), 7875. https://doi.org/10.1038/s41467-025-62828-1.

(7) Formal, F. L.; Pendlebury, S. R.; Cornuz, M.; Tilley, S. D.; Grätzel, M.; Durrant, J. R. Back Electron–Hole Recombination in Hematite Photoanodes for Water Splitting. *J Am Chem Soc* **2014**, *136* (6), 2564–2574. https://doi.org/10.1021/ja412058x.

(8) Ma, Y.; Formal, F. L.; Kafizas, A.; Pendlebury, S. R.; Durrant, J. R. Efficient Suppression of Back Electron/Hole Recombination in Cobalt Phosphate Surface-Modified Undoped Bismuth Vanadate Photoanodes. *J Mater Chem A* **2015**, *3* (41), 20649–20657. https://doi.org/10.1039/c5ta05826k.

(9) He, T.; Benetti, D.; Tseng, C.; Moss, B.; Teschner, D.; Jones, T. E.; Kafizas, A.; Grätzel, M.; Piccinin, S.; Durrant, J. R. Observation of Transition from Rate Law to Butler–Volmer Controlled Water Oxidation Kinetics on Hematite Photoanodes. *J. Am. Chem. Soc.* **2026**, *148* (5), 4833–4838. https://doi.org/10.1021/jacs.5c18734.

(10) Barroso, M.; Mesa, C. A.; Pendlebury, S. R.; Cowan, A. J.; Hisatomi, T.; Sivula, K.; Grätzel, M.; Klug, D. R.; Durrant, J. R. Dynamics of Photogenerated Holes in Surface Modified α-Fe2O3 Photoanodes for Solar Water Splitting. *Proc National Acad Sci* **2012**, *109* (39), 15640–15645. https://doi.org/10.1073/pnas.1118326109.

(11) Li, J.; Wan, W.; Triana, C. A.; Chen, H.; Zhao, Y.; Mavrokefalos, C. K.; Patzke, G. R. Reaction Kinetics and Interplay of Two Different Surface States on Hematite Photoanodes for Water Oxidation. *Nat. Commun.* **2021**, *12* (1), 255. https://doi.org/10.1038/s41467-020-20510-8.

(12) Bassi, P. S.; Xianglin, L.; Fang, Y.; Loo, J. S. C.; Barber, J.; Wong, L. H. Understanding Charge Transport in Non-Doped Pristine and Surface Passivated Hematite (Fe 2 O 3) Nanorods under Front and Backside Illumination in the Context of Light Induced Water Splitting. *Phys. Chem. Chem. Phys.* **2016**, *18* (44), 30370–30378. https://doi.org/10.1039/c6cp05379c.

(13) Cesar, I.; Sivula, K.; Kay, A.; Zboril, R.; Grätzel, M. Influence of Feature Size, Film Thickness, and Silicon Doping on the Performance of Nanostructured Hematite Photoanodes for Solar Water Splitting. *J. Phys. Chem. C* **2009**, *113* (2), 772–782. https://doi.org/10.1021/jp809060p.

(14) Zhong, D. K.; Sun, J.; Inumaru, H.; Gamelin, D. R. Solar Water Oxidation by Composite Catalyst/α-Fe2O3 Photoanodes. *J. Am. Chem. Soc.* **2009**, *131* (17), 6086–6087. https://doi.org/10.1021/ja9016478.

(15) Liu, Y.; Formal, F. L.; Boudoire, F.; Guijarro, N. Hematite Photoanodes for Solar Water Splitting: A Detailed Spectroelectrochemical Analysis on the pH-Dependent Performance. *ACS Appl. Energy Mater.* **2019**, *2* (9), 6825–6833. https://doi.org/10.1021/acsaem.9b01261.

(16) Llorente, V. B.; Jenewein, K. J.; Bierling, M.; Körner, A.; Hutzler, A.; Kormányos, A.; Cherevko, S. Photocorrosion of Hematite Photoanodes in Neutral and Alkaline Electrolytes. *J. Phys. Chem. C* **2023**, *127* (39), 19687–19697. https://doi.org/10.1021/acs.jpcc.3c02969.
